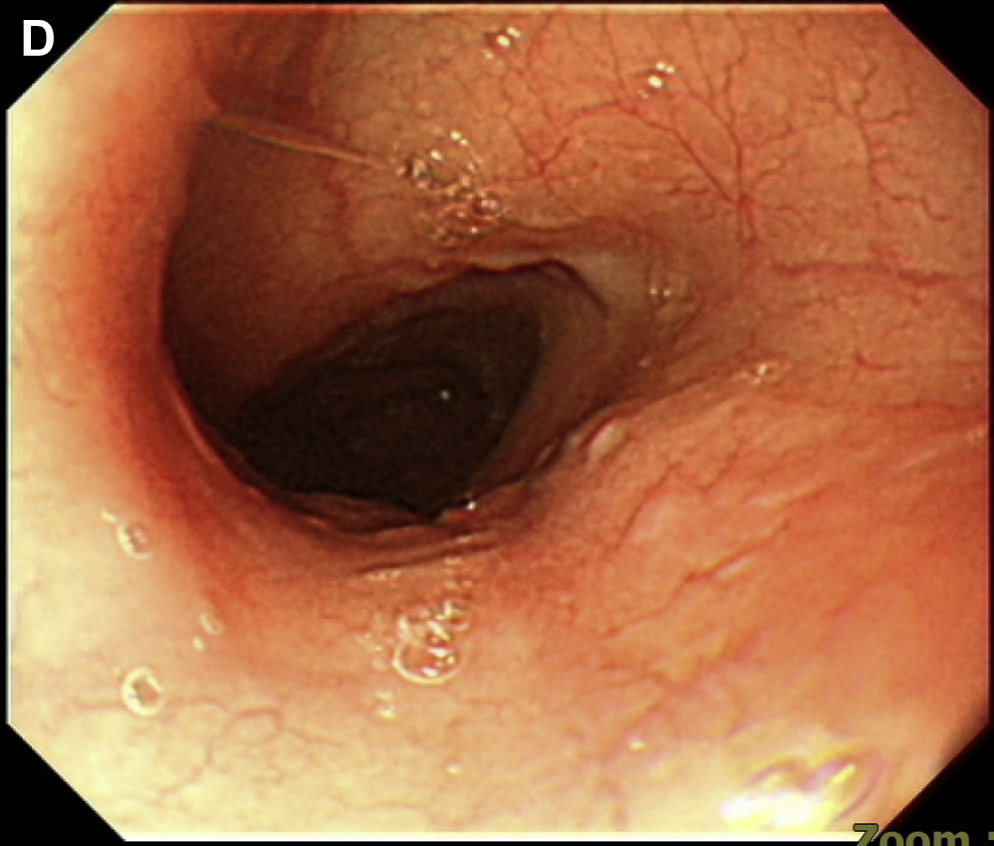# Bleeding Esophagus With Peeling-off Mucosa

**DOI:** 10.1016/j.gastha.2022.04.006

**Published:** 2022-04-20

**Authors:** Hsueh-Chien Chiang, Xi-Zhang Lin

**Affiliations:** Department of Internal Medicine, National Cheng Kung University Hospital, College of Medicine, National Cheng Kung University, Tainan, Taiwan

An 82-year-old woman with a stage 5 chronic kidney disease presented to the hospital with a 2-day history of dyspnea and chest tightness. The physical examination showed bilateral lung edema. For fluid overload, she accepted emergent hemodialysis.

However, her chest tightness persisted, and an episode of hematemesis occurred. An esophagogastroduodenoscopy demonstrated diffuse bleeding esophagitis with peeling-off mucosa at the lower third esophagus (Figure A; Video). Esophageal biopsy was performed at a less-bleeding margin, which revealed esophageal mucosa with necrotic debris, fibrinous exudate, and infiltration of inflammatory cells, compatible with acute esophageal necrosis (AEN) (Figure B). The ECG revealed anterior-lateral wall myocardial ischemic change. She accepted percutaneous coronary intervention for coronary stent insertion at the left anterior descending artery (Figure C). We administered intravenous esomeprazole 40 mg twice daily. Her chest pain improved, and the follow-up esophagogastroduodenoscopy showed recovered esophageal mucosa (Figure D) 1 week later.

AEN indicates diffuse esophagitis and circumferential hemorrhagic mucosal peeling at the lower third esophagus. AEN occurs mostly in old patients with a severe illness, including hypoperfusion, ischemia, and severe sepsis. In our patient, we discovered the etiology of AEN as ischemic heart-related hypoperfusion. After the coronary artery stenting, the AEN recovered dramatically. Identifying and solving the etiology of hypoperfusion is important in managing patients with AEN.

**Figure:**
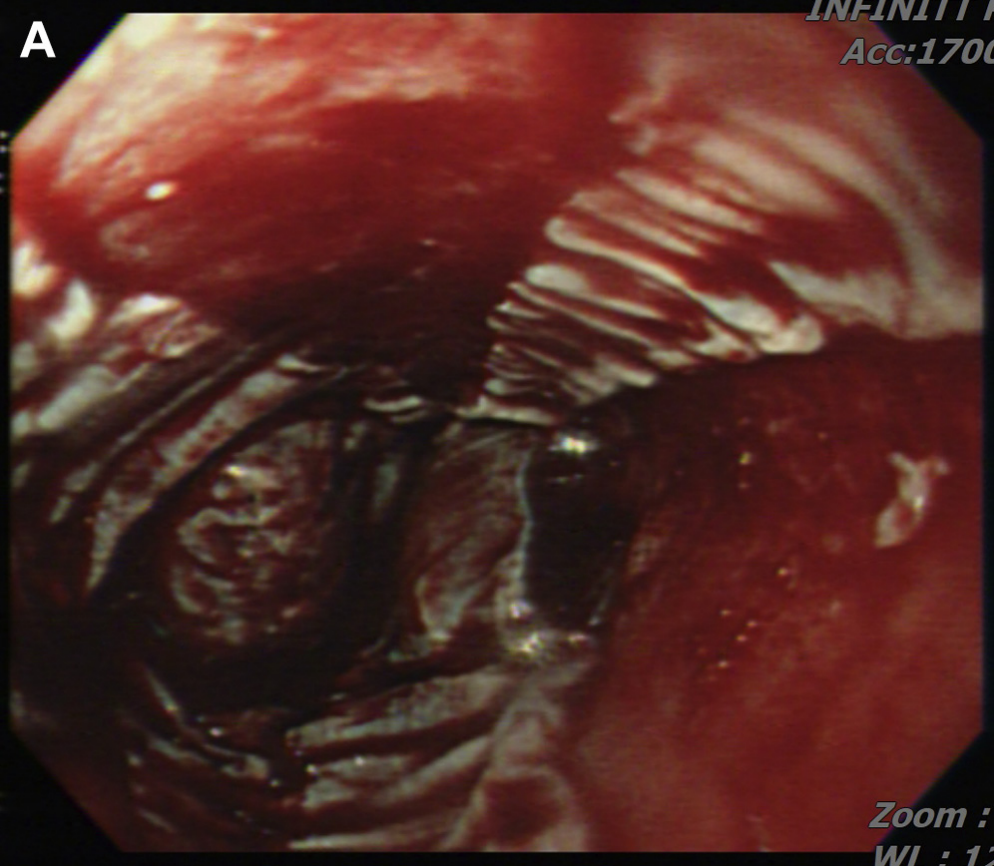

**Figure:**
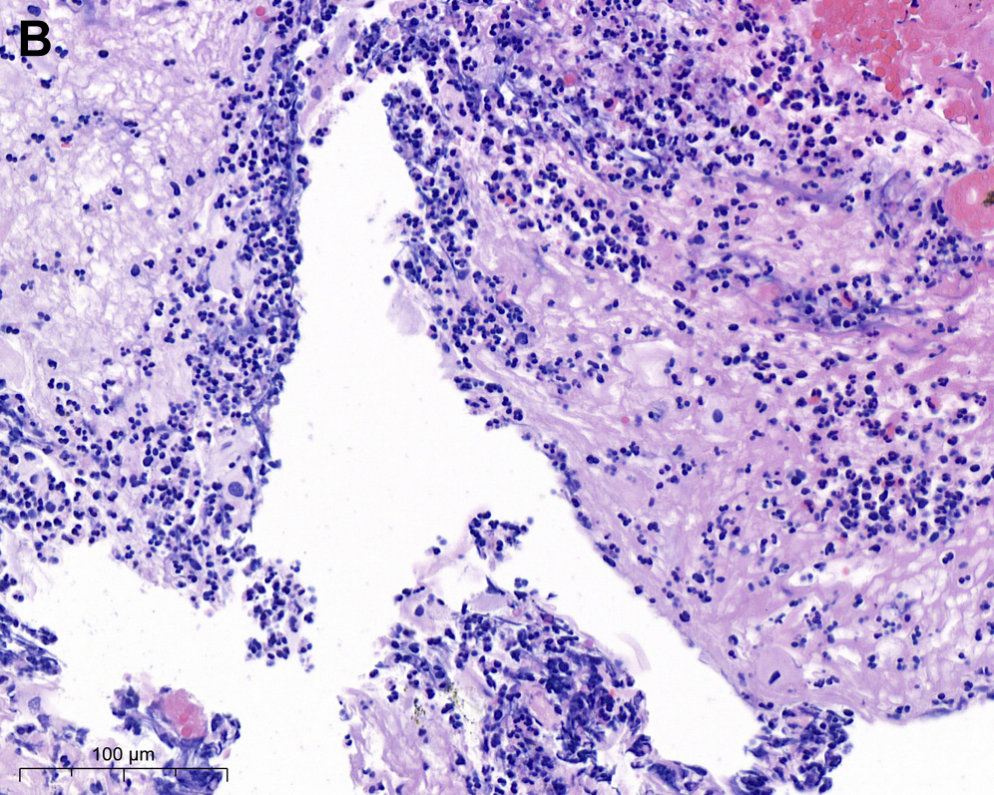

**Figure:**
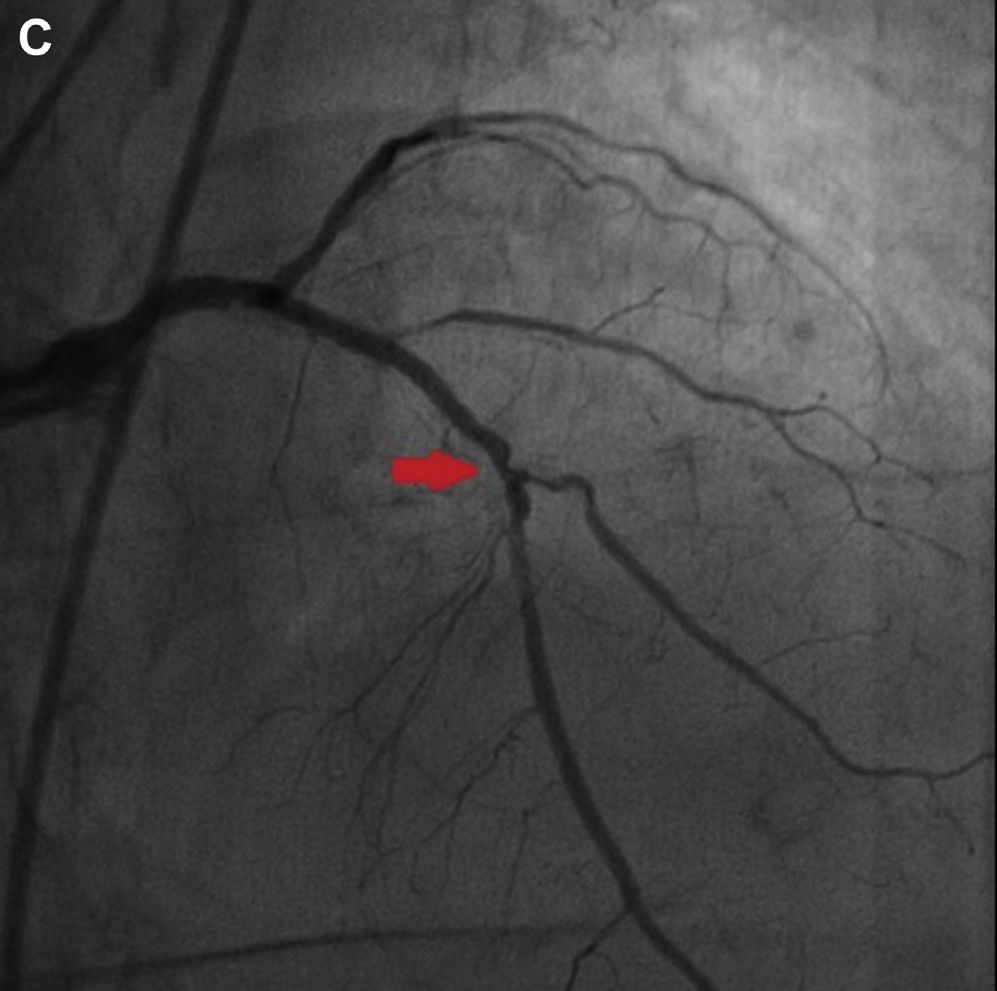

**Figure:**